# Syndecan-1 as a predictor of vulnerable atherosclerotic plaques

**DOI:** 10.3389/fcell.2024.1415788

**Published:** 2024-08-08

**Authors:** Yan Qiu, Zhi Ouyang, Jian Zhong, Linlu Jin, Yixue Qin, Ye Zeng

**Affiliations:** ^1^ Institute of Biomedical Engineering, West China School of Basic Medical Sciences and Forensic Medicine, Sichuan University, Chengdu, China; ^2^ Department of Cardiovascular Surgery, Fuwai Yunnan Cardiovascular Hospital, Kunming, China

**Keywords:** atherosclerosis, vulnerable plaque, glycocalyx, syndecan-1, sphingosine 1-phosphate

## Abstract

**Aims:**

Cardiovascular disease remains a major global health concern, with atherosclerosis (AS) being a significant contributor. Vulnerable plaques play a critical role in acute cardiovascular events. Syndecan-1 (SDC-1), a vital membrane proteoglycan in the vascular endothelial glycocalyx, is believed to be associated with plaque progression. However, its precise relationship with severity and vulnerability of atherosclerotic plaque remains unclear. This study aimed to investigate SDC-1 expression and its potential correlation with plaque vulnerability in ApoE^−/−^ atherosclerosis mouse model.

**Methods and results:**

Eight-week-old mice were induced into the AS model using a high-fat diet (HFD) and/or partial ligation of the left common carotid artery (PLCA), with a chow diet (CD) control group. After 16 weeks, plaques in the aortic root showed the following order: HFD + PLCA group > HFD group > CD + PLCA group > CD group. Immunohistochemistry revealed heightened accumulation of lipid/foam cells and CD68-labeled macrophages in the plaques, elevated vascular endothelial growth factor (VEGF), and matrix Metalloproteinase-9 (MMP-9) in the HFD + PLCA group’s plaques, along with reduced collagen and α-SMA-labeled smooth muscle cells, resulting in the highest vulnerability index value. Immunohistofluorescence analysis of frozen plaque sections showed significantly higher SDC-1 expression in the AS mice group compared to the CD group, both positively correlated with plaque vulnerability. Serum analysis demonstrated elevated levels of SDC1, sphingosine 1-phosphate (S1P), and VEGF-A in the AS mice, all positively correlated with plaque vulnerability. Multivariate analysis identified SDC1 as an independent predictor of plaque vulnerability.

**Conclusion:**

This study enhances our understanding of plaque vulnerability mechanisms and presents SDC1 as a potential biomarker for atherosclerosis. These findings underscore the importance of addressing modifiable risk factors, such as diet and hemodynamics and suggest the utility of serum SDC1 as a valuable clinical marker. Ultimately, these insights may lead to more effective strategies in combating cardiovascular diseases and improving patient outcomes.

## 1 Introduction

The latest global burden of disease data underscores the alarming impact of cardiovascular disease, which caused approximately 18.6 million deaths worldwide in 2019, surpassing infectious diseases as the leading cause of death and disability (Roth et al., 2020). Atherosclerosis (AS) significantly contributes to the prevalence of cardiovascular disease, characterized by lipid deposition and chronic inflammation in the arterial wall, making it the primary underlying pathology for cardiovascular conditions (Kobiyama and Ley, 2018).

The concept of vulnerable plaques, first introduced in 1989 (Muller et al., 1989), pertains to atherosclerotic plaques that pose a high risk of cardiovascular events (Ino et al., 2011). Research indicates that a substantial percentage (60%–80%) of acute coronary syndromes (ACS) arise from plaque rupture without significant lumen stenosis, with vulnerable plaques being the primary instigator (Flaherty et al., 2013). Identifying and assessing potentially vulnerable plaques before acute cardiovascular events occur is of paramount important in clinical practice, as it holds the key to preventing or mitigating acute coronary events and sudden cardiac death. The exact cause of plaque rupture remains elusive, but certain features such as large lipid nuclei, bleeding, inflammatory cells, and thin fibrous caps have been associated with ruptured or high-risk plaques (John Chapman and Preston Mason, 2022). Due to the insufficiency of each feature alone in identifying high-risk plaques, a comprehensive approach entails utilizing a combination of features. As a result, experimental studies on atherosclerotic plaques employ the vulnerability index (VI), which calculates the ratio between unstable and stable plaque components (Erlöv et al., 2016; Tomas et al., 2018). The underlying principle of VI is to capture the delicate balance between factors that destabilize plaques (macrophages, bleeding, and lipids) and those that stabilize them (smooth muscle cells and collagen), as these factors ultimately determine the likelihood of plaque rupture. Collagen, primarily synthesized and secreted by smooth muscle cells (SMCs), plays a critical role in plaque stability. Enhanced migration and proliferation of SMCs contribute to plaque stability, whereas SMC apoptosis leads to plaque instability (Grootaert et al., 2018). Matrix metalloproteinases (MMPs) and cathepsin can degrade collagen components, weakening fibrous caps (Libby et al., 1998). Therefore, measuring the vulnerability of a plaque can also involve considering the ratio of collagen degradation-related enzymes, collagen, SMCs, and apoptotic SMCs (Bennett, 1999).

Early attempts to quantify plaque vulnerability gave rise to the “vulnerability index” proposed by Shiomi et al. (2001), relying on the ratio between the sum of extracellular lipids (%) and macrophages (%) and the sum of smooth muscle cells (%) and collagen fibers (%) to assess plaques. However, practical application revealed that the formula had not encompass key vulnerable elements of plaques, such as vascular endothelial growth factor (VEGF) (Fittipaldi et al., 2016) and matrix metalloproteinase-9 (MMP-9) (Li et al., 2020), rendering it unsuitable for complex lesions and necessitating further optimization.

The glycocalyx (GCX) is a complex layer of polysaccharide-protein that covers the lateral surface of the endodermal cavity of blood vessels. Within the endothelial GCX, membrane-bound proteoglycans, composed of glycosaminoglycans attached to core proteins, play a vital role in maintaining vascular system homeostasis and influencing its function (Miranda et al., 2016). Syndecan-1 (SDC1) is a prominent constituent of the GCX (Möckl, 2020). As a transmembrane hybrid proteoglycan, SDC1 carries chondroitin sulfate and heparin sulfate chains, providing a protective cover to endothelial cells and facilitating their connection with the surrounding environment (Weinbaum et al., 2021).

The development of atherosclerosis involves multiple factors, and damage to the GCX is considered a crucial underlying cause of cholesterol accumulation on the blood vessel wall, ultimately leading to plaque formation. Studies, such as the work of Tsiantoulas et al., 2021, have demonstrated that intact acetyl heparan sulfate glycoproteins (HSPGs) play a protective role, preventing the formation of atherosclerotic plaques by interacting with A proliferation-inducing ligands (APRIL). When the structural integrity of the GCX is compromised, the vascular endothelium may suffer damage. Low-density lipoprotein (LDL) passes through the thin GCX, enters the inner lining of the artery, and becomes oxidized, triggering inflammation and initiating the process of plaque formation (Cancel et al., 2016). Thus, the GCX holds promise as a potential marker for cardiovascular and cerebrovascular diseases, such as atherosclerosis, facilitating their early diagnosis (Xue et al., 2018). In fact, our understanding of *in vivo* changes in the GCX and its relationship with plaque vulnerability under atherosclerosis conditions remains incomplete. Recent evidence suggested that disruption of endothelial GCX is associated with the development of atherosclerosis, and that GCX components are shed into the blood stream (Ahn et al., 2023). However, it was reported that lower serum levels of SDC1 (<99.0 ng/mL) are associated with a higher prevalence of lipid-rich plaques in patients with coronary artery diseases (CADs) (Nemoto et al., 2020). Therefore, the GCX presents an intriguing avenue for researchers to combat cardiovascular diseases, but further evidence is required to ascertain its specific role in such diseases. Advanced imaging techniques are also needed to directly visualize the GCX *in vivo*, enabling the establishment of a comprehensive “GCX function” diagnosis and treatment system in clinical practice, thus fostering the development of cardiovascular and cerebrovascular disease prevention and treatment.

Sphingosine 1-phosphate (S1P) is a signaling lipid synthesized by sphingosine kinases (SphK1 and SphK2) that exerts diverse effects on cardiovascular function. Studies have revealed that S1P primarily exerts anti-atherogenic effects through its action on S1P receptors 1 and 3 (S1PR1 and S1PR3), while it may have pro-atherogenic effects via S1PR2 (Kurano and Yatomi, 2018). Notably, Ishimaru et al. (2019) demonstrated the significant role of SphK2 in the formation of atherosclerotic plaques, as evidenced by larger atherosclerotic lesions observed in mice with the SphK2 gene deleted compared to control mice. Additionally, Keul et al. (2022) recently highlighted that treatment with 4-deoxypyridoxine (DOP) accelerated the development of atherosclerosis in a cholesterol-fed ApoE^−/−^ mouse model, leading to the predominant formation of an unstable plaque phenotype characterized by frequent plaque rupture and atherosclerotic thrombosis.

Previous studies have suggested that S1P functions as a vasoactive factor, playing a protective role in maintaining the integrity of the endothelial GCX (Zeng et al., 2014). However, the specific changes of S1P in the context of atherosclerosis are still a subject of debate.

Nevertheless, the relationship between these changes and S1P, as well as the specific roles of S1P and the GCX in the development of vulnerable atherosclerotic plaques, remain unclear. Therefore, the aim of this study was to develop an advanced quantitative assessment system for vulnerable plaque in atherosclerotic apolipoprotein E-deficient (ApoE^−/−^) mice. Our investigation focused on analyzing the expression of the GCX and S1P in plaques and serum to determine their potential correlation with plaque vulnerability. By shedding light on the interplay between S1P, the GCX, and vulnerable atherosclerotic plaques, our research endeavors to contribute valuable insights to the understanding of atherosclerosis pathogenesis and may pave the way for novel diagnostic and therapeutic approaches in the management of cardiovascular diseases.

## 2 Materials and methods

### 2.1 Animal experiments

All animal experiments conformed to the guidelines from Directive 2010/63/EU of the European Parliament on the protection of animals used for scientific purposes and were approved by the Animal Experimentation Ethics Committee of Sichuan University (No. K2021013). All procedures involving animals were performed under isoflurane anesthesia, and every effort was made to minimize pain and discomfort.

Male wild-type C57BL/6 mice and C57BL/6-ApoE^−/−^ mice were procured from Byrness Weil biotech Ltd. and subsequently bred in-house. Genotyping was performed to confirm the ApoE deficiency in the mice. Throughout the experimental period, the animals were housed under specific conditions, including a 12-h light-dark cycle, relative humidity between 40%–70%, and a room temperature (RT) maintained within the range of 20°C–25°C. The experimental groups were as follows: after an acclimatization period, mice at 8 weeks of age underwent partial ligation of the left carotid artery (PLCA), and then fed either a normal diet (CD + PLCA) or a high-fat diet (HFD + PLCA). The control groups, underwent the same surgical procedure without ligation, consisted of ApoE^−/−^ mice fed either a normal diet (Sham) or a high-fat diet (HFD), as well as C57BL/6 mice fed a normal diet (Wild Type CD). During the experimental period of 16 weeks, the mice had unrestricted access to food (procured from Byrness Weil biotech Ltd. Co., Ltd.) and tap water.

### 2.2 Partial ligated carotid artery (PLCA)

**FIGURE 1:**
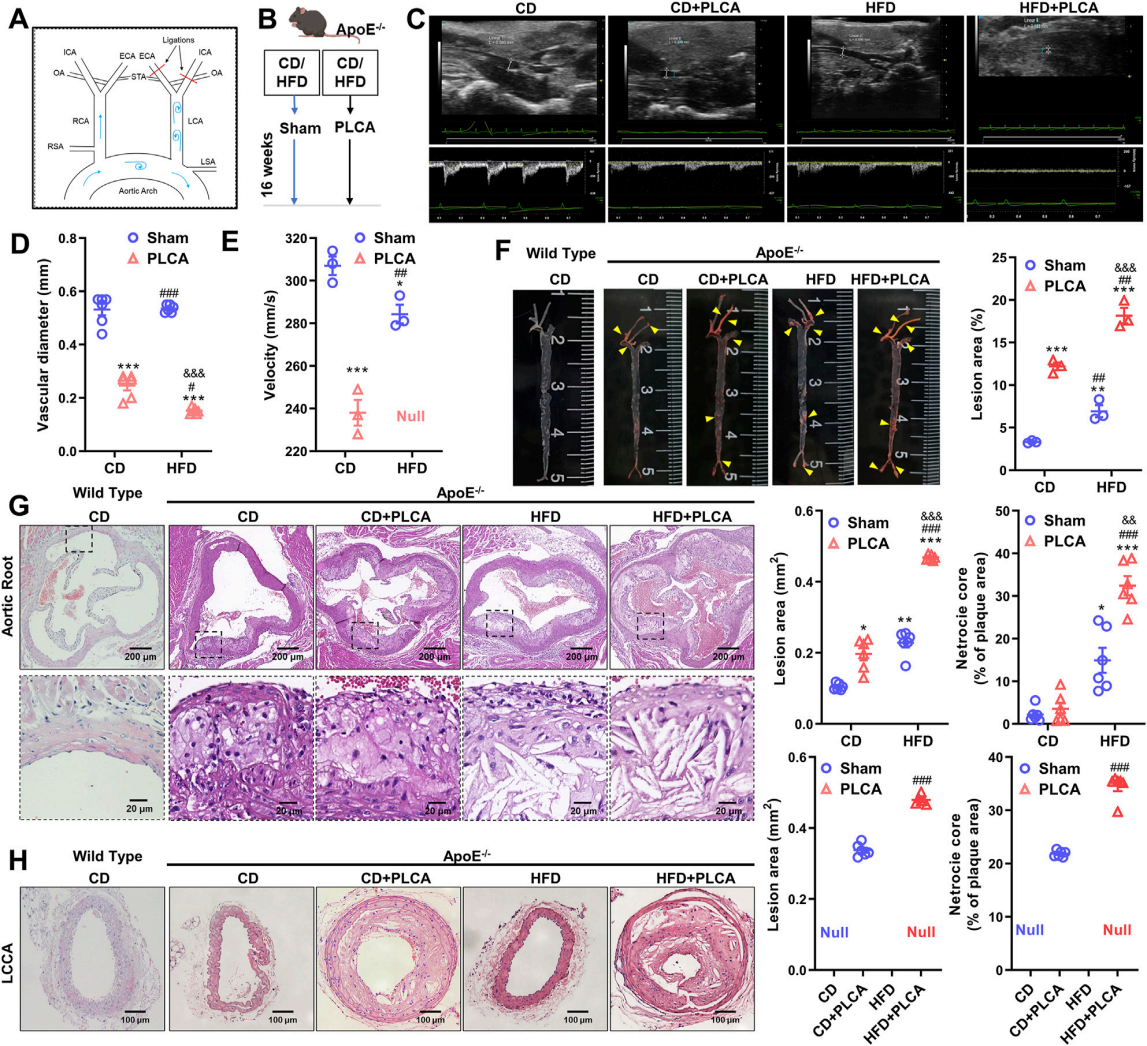
Description of the mouse model and analysis of structural features of atherosclerotic plaques. **(A)** Schematic diagram illustrating the partial ligation of the carotid artery. Three out of the four caudal branches of the left carotid artery were ligated, while the superior thyroid artery was preserved. **(B)** Experimental grouping of the study. **(C)** After 16 weeks of modeling, small animals underwent ultrasonographic examination to assess plaque morphology and blood flow in the left common carotid artery of ApoE^−/−^ mice (n = 3). **(D–E)** Statistical plots presenting quantitative analysis of vessel diameter and blood flow velocity. Null indicates that no blood flow velocity was detected, one-way ANOVA. **(F)** Oil red O staining was employed to detect lipid deposition in aortic plaques from C57BL/6 and ApoE^−/−^ mice; yellow arrows indicate stained plaques (n = 3, one-way ANOVA). **(G)** HE staining was performed to assess the morphology of aortic root plaques (n = 6, one-way ANOVA). The scale bar represents 200 μm, and the scale bar in the dashed box represents 20 μm. **(H)** HE staining of the morphology of the left common carotid artery plaque (LCCA) (n = 6, unpaired *t*-test). The scale bar is 100 μm *P < 0.05, *p < 0.01, ***P < 0.001, compared with CD group; ^#^ *P* < 0.05, ^##^ *P* < 0.01, ^###^ *P* < 0.001, compared with CD + PLCA group; ^&&^ *P* < 0.01, ^&&&^ *P* < 0.001, compared to HFD group.

Prior to PLCA procedure, ApoE^−/−^ mice were fed a normal chow diet and water. The PLCA procedure was conducted following a previously described method (Sullivan and Hoying, 2002), with slight modifications. Briefly, anesthesia was induced using isoflurane inhalation at a dose of 3%. The surgical area was disinfected with iodine, and a midline incision was made ventral to the neck. The left carotid artery (LCA) was exposed through blunt dissection. Three of the four caudal branches of the LCA, namely, the left external carotid artery (ECA), internal carotid artery (ICA), and occipital artery (OA), were ligated using 6–0 silk sutures, while the superior thyroid artery (STA) was left intact (Figure 1A). The incision was then closed using absorbable sutures. The control group underwent the same surgical procedure without ligation after blunt separation. Post-surgery, the mice were closely monitored in a room equipped with a heating pad until they fully recovered. Following the partial ligation, ApoE^−/−^ mice were maintained on either a high-fat diet or a regular diet until the end of the study, which ranged from 2 days to 16 weeks.

### 2.3 Plasma lipid measurement

Orbital blood samples were collected from the mice after isoflurane anesthesia into tubes without anticoagulants. The collected blood samples were then centrifuged at 4°C for 15 min at 3,000 rpm. The supernatant was collected for lipid assays, including total cholesterol (TC), triglycerides (TG), high-density lipoprotein (HDL), low-density lipoprotein (LDL), and very low-density lipoprotein (VLDL). These lipid measurements were performed using a fully automated biochemistry instrument (AU480, Beckman Coulter, United States).

### 2.4 Ultrasound molecular imaging of atherosclerotic common carotid artery

The Visual-Sonics Vevo 3100 ultra-high-resolution small animal ultrasound imaging system (Fujifilm Visualsonics, Japan) was utilized for observing plaque formation in the left common carotid artery (LCCA) plaque of AS mice.

(1) Animal preparation: ApoE^−/−^ mice were placed in the induction chamber of a gas anesthesia machine, and the isoflurane concentration was adjusted to 2%. After induction, continuous inhalation of isoflurane through a face mask was used to maintain anesthesia. The fur on the neck and chest of the mice was removed using hair removal cream. The mice were then positioned supine on the operating table with continuous inhalation of isoflurane through the mouth and nose. The limbs of the mice were kept close to the electrode sheet, and the temperature of the operating table was maintained at a constant 37°C. ECG metal electrodes were covered with conductive paste, and the mice’s ECG was recorded while maintaining a heart rate of 220–420 beats per minute.
(2) Image acquisition: A coupling agent was applied to the neck of the mice, and the MS-500 probe was used to acquire ultrasound and M-ultrasound images. The probe was rotated counterclockwise while keeping it vertical and with the incision facing the animal’s head to obtain long-axis section images. Slight clockwise rotation of the probe was performed to optimize the images. Pulsed-wave Doppler mode was used to measure flow velocity at the entrance, midpoint, and exit of the common carotid artery. Vessel size was measured using M-mode, and vessel length was measured using B-mode.

### 2.5 Preparation of histological sections

ApoE^−/−^ and C57BL/6 male mice were euthanized after 16 weeks of raising, following overnight fasting without water. After isoflurane inhalation anesthesia, the mice were decapitated, and the entire aorta was isolated. The abdominal region was incised with scissors to remove excess organs, followed by the separation of the heart and kidneys. Excess fat was removed from the periphery to expose the thoracoabdominal aorta. The aorta was carefully separated under a body vision microscope, and the peripheral fatty tissue and connective tissue were removed. Subsequently, the aorta, along with the attached heart, was placed in EP tubes containing 4% paraformaldehyde solution for 24–48 h. The bilateral common carotid arteries, aortic arch, and aortic root were dissected from the mice and embedded in paraffin. Serial tissue sections were stained with HE, Masson’s trichrome, Sirius Red, immunohistochemistry, and immunofluorescence to observe plaque formation and evaluate plaque stability.

### 2.6 Quantitative analysis of lesion composition

The area of macrophages, smooth muscle cells, extracellular lipid deposition, and lesion area were measured following a previously reported method (Centa et al., 2019). Smooth muscle cells were defined as cells stained with Alpha-smooth muscle actin (α-SMA), macrophages were defined as cells stained with CD68, and extracellular lipid deposits were identified as vacuoles and lacunae in the staining (Hartwig et al., 2015). Collagen fibers were identified using sections stained with Picrosirius red and Masson’s trichrome (Edsfeldt et al., 2022).

### 2.7 Histology, immunohistochemistry and immunofluorescence

Aortic root and LCCA plaque samples used for histopathologic examination were fixed in 4% paraformaldehyde, dehydrated, paraffin-embedded, and sectioned at 5 µm thickness. Sections were then stained for neutral lipids (Oil Red O), macrophages (CD68), smooth muscle cells (α-actin), angiogenesis within the plaque (VEGF), and collagen (Masson’s trichrome and Sirius Red). CD68, α-SMA and MMP-9 were stained using rabbit anti-human CD68 (1:100, bs-4819R, BIOSS, China), α-SMA (1:100, A1011, Abclonal, China) and MMP-9 (1:100, bs-4593R, BIOSS, China). Following incubation with peroxidase-conjugated anti-rabbit IgG secondary antibodies (1:200, Invitrogen, United States), 3,3′-24 diaminobenzidine (DAB) was used for staining detection and nuclei were counterstained with 25 Mayer’s hematoxylin. Positively stained plaque areas (% of total plaque area) were scanned.

For immunofluorescence staining, 5 µm paraffin sections were cut and blocked for 45 min on RT in PBS containing 5% goat serum. After blocking, sections were incubated with a rabbit anti-human CD68 (1:100, bs-4819R, BIOSS, China), α-SMA (1:100, A1011, Abclonal, China) and mouse anti-human VEGF (1:100, TA500289, Origene, United States of America) antibodies overnight at 4°C. For lipid droplet and SDC1 detection, the cardiac tissue was immediately excised, flattened within an embedding cassette, and rapidly infused with optimal cutting temperature (OCT). The tissue was then quickly frozen for 1–3 min until it, along with the embedding medium, formed a solid, white, icy block. The frozen tissue block was subsequently sectioned into 5 μm slices. Immunofluorescence staining was performed on the cryosections with SDC1 (1:100, sc-390791, Santa, United States) and BODIPY (2 μM, GC42959, GLPBIO, United States) antibodies overnight at 4°C, followed by donkey anti-rabbit Alexa 555 (A0453, Beyotime, China) and goat anti-rabbit Alexa 488 (CA11034S, Invitrogen, United States) antibodies for 1 h on RT (1:100). The sections were then stained with DAPI (D3571, Invitrogen, United States) for 5 min. Confocal microscopy (LSM710, Carl Zeis, Germany) was used to observe the images.

### 2.8 Vulnerability index (VI_t_ and VI_m_)

The Vulnerability Index (VI) was employed to evaluate the overall degree of vulnerability between the models by considering all plaque features analyzed in the study. While the traditional formula proposed by Shiomi et al. (2001) (VI_t_) is widely used as the reference standard for assessing plaque instability, it does not include critical components affecting plaque vulnerability, such as MMP-9 and VEGF, making it not suitable for complex lesions. The area of positive staining of plaque components were analyzed using ImageJ with “threshold” function. Therefore, in this study, the critical components influencing plaque instability were considered together, and six key indicators influencing plaque stability were selected to assess the overall degree of vulnerability between the models (VI_m_).

$${\text{VI}}_{t}=\frac{\text{Lipid}\%+\text{Macrophages}\%}{\text{Smooth}\;\text{muscle}\;\text{cells}\%+\text{Collagen}\;\text{fibers}\%}$$

$${\text{VI}}_{m}=\frac{\text{Lipid}\%+\text{Macrophages}\%+\text{VEGF}\%+\text{MMP}-9\%}{\text{Smooth}\;\text{muscle}\;\text{cells}\%+\text{Collagen}\;\text{fibers}\%}$$

### 2.9 Statistical analysis

Quantitative analysis of the images was performed using ImageJ (v1.53k, NIH, United States of America), and data were plotted using Graphpad Prism (v9.3.1, Dotmatics, United States). Experimental data were expressed as mean ± standard deviation (SD). Statistical analysis was performed by one-way ANOVA with either the least significant difference (LSD) test or Tamhane’s T2 test (depending on Levene’s statistic for homogeneity of variance), using the SPSS 26.0 software package (IBM, United States). Pearson correlation analysis was used to assess the correlation between GCX in plaques and plaque vulnerability index. Furthermore, binary logistic regression was conducted to determine whether serum SDC1, S1P, and VEGF-A were independent predictors of plaque vulnerability. *p* < 0.05 was considered statistically significant.

## 3 Results

### 3.1 Mouse model description and analysis of atherosclerotic plaque structural features

In this study, we modeled atherosclerosis (AS) in mice by subjecting the mice to a high-fat diet (HFD) and/or partially ligating the left common carotid artery (PLCA) (n = 6), while the control group was fed a normal chow diet (CD) (n = 6). The modeling period lasted for 16 weeks (Figures 1A,B). Doppler ultrasound was used for clinical detection and evaluation of occlusive carotid artery disease. The degree of luminal stenosis was considered an important indicator of the risk of atherosclerotic burden. Additionally, plaque morphology plays a crucial role in assessing plaque vulnerability.

We observed a significant reduction in the lumen diameter of the left common carotid artery in the AS group compared to the CD group, with the HFD + PLCA group showing the most significant reduction (*p* < 0.001). The intravascular flow velocity was significantly lower in the AS group (*p* < 0.05) (Figures 1C–E). After 16 weeks of high-fat chow feeding, the mice were dissected, and the entire aorta was obtained. Plaque formation in the overall aortic wall was detected using oil red O staining (Figure 1F).

No significant plaque formation was observed in the aorta after 16 weeks of feeding C57BL/6 mice with a normal chow diet. Atherosclerotic plaques were more prominent in the CD + PLCA, HFD, and HFD + PLCA groups of ApoE^−/−^ mice compared to the CD group. In the surgically ligated group at the left carotid artery, significant plaques were visible, almost filling the entire carotid artery. The plaque area/aortic area was 3.33% ± 0.12%, 12.20% ± 0.03%, 6.93% ± 0.99%, and 18.16% ± 1.29% in the CD and CD + PLCA, HFD, and HFD + PLCA groups, respectively. The plaque area was significantly higher in the AS model group compared to the control group, with the most significant plaque formation in the HFD + PLCA group (*p* < 0.001). HE staining of the aortic roots showed that after 16 weeks of normal diet feeding, no significant plaques were formed in the aortic roots of C57BL/6 mice, while a large number of plaques were visible in the aortic root sites of ApoE^−/−^ (Figures 1G,H).

The plaques in the HFD and HFD + PLCA groups exhibited a large number of foam cells and cholesterol crystals with thin fibrous caps, while the CD and CD + PLCA groups had fewer foam cells and cholesterol crystals. The results showed a significant increase in plaque area in the CD + PLCA group (*p* < 0.05), HFD group (*p* < 0.01), and HFD + PLCA group (*p* < 0.001), as well as a significant increase in lipid core area in the HFD group (*p* < 0.05) and HFD + PLCA group (*p* < 0.001) compared to the CD group. The plaque area size and lipid core area were significantly increased in the HFD + PLCA group compared to the CD + PLCA group and the HFD group (*p* < 0.001). There was no statistically significant difference in lesion area and lipid core area between the HFD group and the CD + PLCA group. However, the lesion area and lipid core area were significantly increased in the HFD + PLCA group (*p* < 0.001). These results indicate that the atherosclerosis mouse model was successfully created, and this AS mouse model can be used for subsequent evaluation of plaque vulnerability. The findings show that there was no significant difference in plaque area between the aortic root and LCCA in the HFD + PLCA group (0.47 mm^2^ vs. 0.48 mm^2^, *p* > 0.05), while compared to the aortic root in the CD + PLCA group, the plaque area was considerably greater in the LCCA group (0.20 mm^2^ vs. 0.34 mm^2^, *p* < 0.001).

### 3.2 Plaque composition

#### 3.2.1 Destabilizing components

**FIGURE 2:**
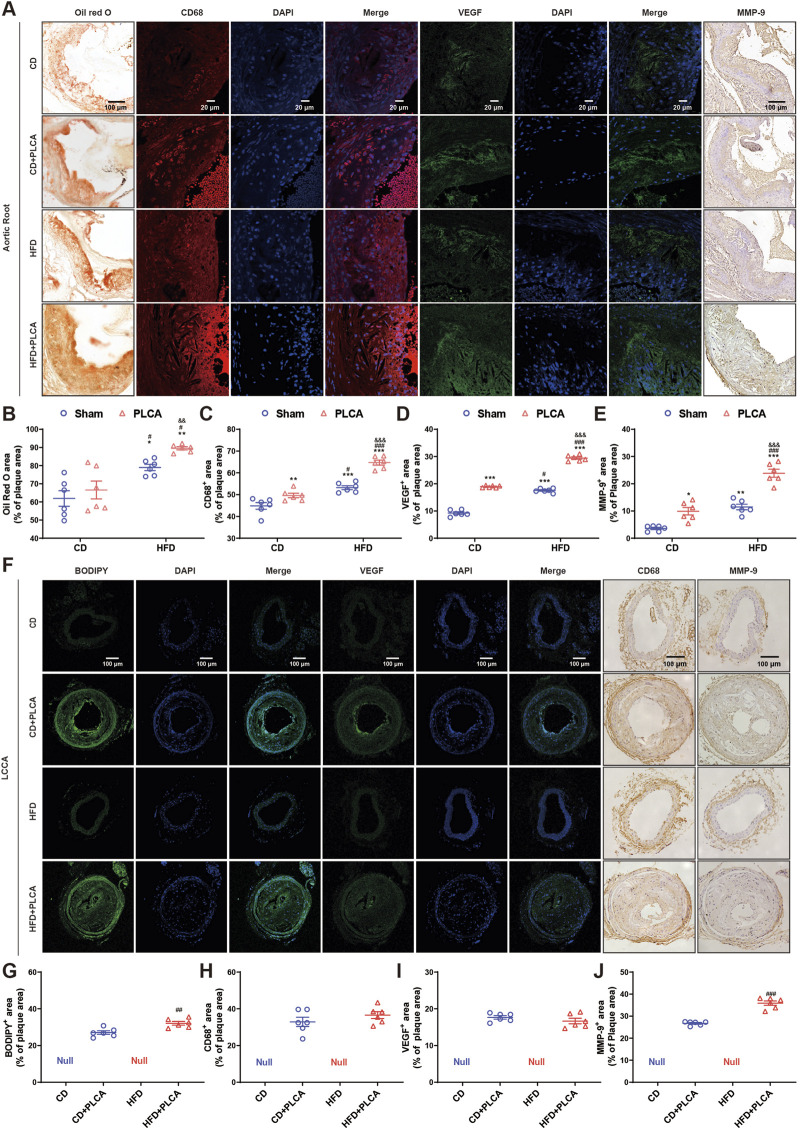
Analysis of destabilizing components in atherosclerotic plaque. **(A)** The expression of lipid deposition in aortic root plaques was detected by oil red O with a scale bar of 100 μm, immunofluorescence detection of CD68 expression (macrophage marker) and VEGF in aortic root plaques with a scale bar of 20 μm, and IHC detection of MMP-9 expression in aortic root plaques with a scale bar of 100 μm. **(B–E)** Statistical chart for quantitative analysis of oil red o staining, CD68, VEGF and MMP-9 in the aortic root plaques (n = 6, one-way ANOVA). **(F)** Immunofluorescence detection of BODIPY and VEGF expression in LCCA plaques with a scale bar of 100 μm. IHC detection of CD68 and MMP-9 expression in LLCCA plaques with a scale bar of 100 μm. **(G–J)** Statistical chart for quantitative analysis of BODIPY staining, VEGF, CD68 and MMP-9 in the LCCA plaques (n = 6, unpaired *t*-test). *P < 0.05, **P < 0.01, ***P < 0.001, compared with CD group; ^#^ *P* < 0.05, ^##^ *P* < 0.01, ###*P* < 0.001, compared with CD + PLCA group; ^&&^ *P* < 0.01, ^&&&^ *P* < 0.001, compared to HFD group.

An analysis was conducted on various components such as lipids, macrophage content, VEGF, and MMP-9, all of which influence plaque instability (Figures 2A,F). Compared with the CD group, no significant change was observed in lipid deposition in the CD + PLCA group, while there was an 11% increase in macrophage infiltration in the aortic root plaques (*p* < 0.01). The HFD group exhibited a 28% increase in extracellular lipid deposition (*p* < 0.05) and a 20% increase in macrophages (*p* < 0.001). In comparison, the HFD + PLCA group showed a 45% increase in lipid accumulation (*p* < 0.01) and a 44% increase in macrophage infiltration (*p* < 0.001) in plaques (Figures 2B, C).

When compared with the CD + PLCA group, the HFD group showed a significant increase in extracellular lipid deposition by 19% (*p* < 0.05) and macrophage infiltration by 7% (*p* < 0.05). The HFD + PLCA group demonstrated a significant increase in extracellular lipid deposition by 34% (*p* < 0.01) and macrophage infiltration by 30% (*p* < 0.001). Comparing the HFD + PLCA group with the HFD group, there was a 13% increase in extracellular lipid deposition (*p* < 0.01) and a 22% increase in macrophage infiltration (*p* < 0.001) (Figures 2B, C).

Intraplaque neovascularization, characterized by the overexpression of VEGF, is a risk factor for plaque rupture (Steinkamp et al., 2021). Therefore, VEGF staining of aortic root plaques was performed to identify intraplaque neovascularization (Figure 2D). Compared with the CD group, VEGF expression was significantly increased in aortic root plaques in the other three groups (*p* < 0.001). VEGF expression was significantly elevated by 55% in plaques in the HFD + PLCA group compared with the CD + PLCA group (*p* < 0.001). It was significantly increased by 68% in the HFD + PLCA group compared with the HFD group (*p* < 0.001).

MMP-9, also known as gelatinase B, is a member of the MMP family that is closely associated with plaque instability (Myasoedova et al., 2018). MMP-9 is highly expressed in the fibrous cap and lipid core of atherosclerotic plaques, and it is more abundant and active in unstable plaques (Brown et al., 2017). MMP-9 staining of aortic root plaques was performed (Figure 2E), revealing a significant increase in MMP-9 expression in the CD + PLCA group by 175% (*p* < 0.05), the HFD group by 219% (*p* < 0.01), and the HFD + PLCA group by 563% (*p* < 0.001) compared to the CD group.

MMP-9 expression within the plaques was significantly increased by 140% in the HFD + PLCA group compared to the CD + PLCA group (*p* < 0.001), and the difference between the HFD and CD + PLCA groups was not significant. It was significantly elevated by 108% in the HFD + PLCA group compared to the HFD group (*p* < 0.001).

BODIPY 493/503, a fluorescent neutral lipid dye, was used for microscopic observation of lipid droplets (LDs) (Figures 2F,G). The HFD + PLCA group demonstrated a significant increase in lipid accumulation compared to the CD + PLCA group (*p* < 0.01), with no significant difference in macrophage infiltration. Although no significant change in CD68 and VEGF expression was observed (Figures 2H, I), MMP-9 expression was significantly higher in LCCA plaques in the HFD + PLCA group compared to the CD + PLCA group (*p* < 0.001) (Figure 2J).

#### 3.2.2 Stabilized components

**FIGURE 3:**
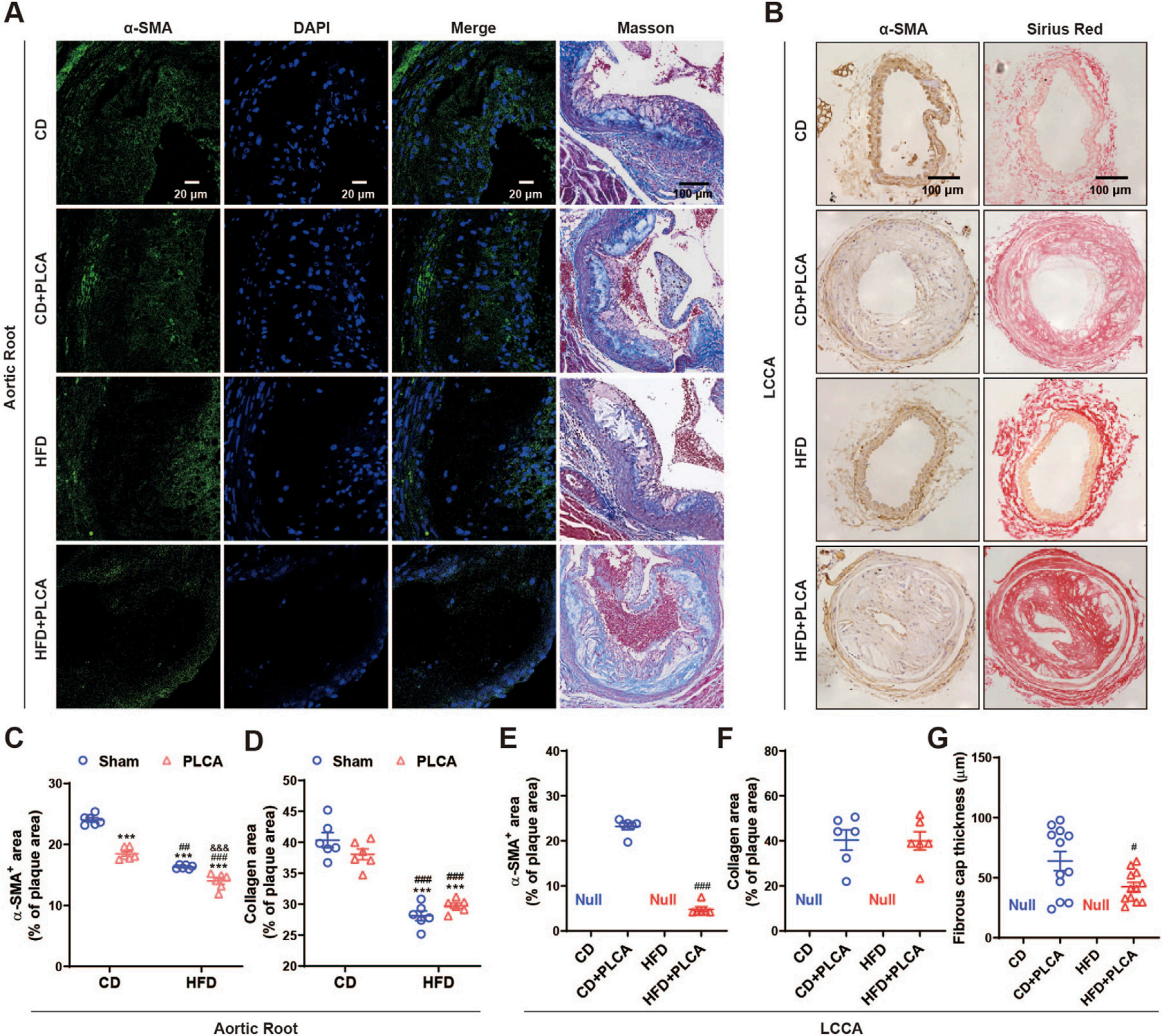
Examination of stable components in atherosclerotic plaque. **(A)** Immunofluorescence detection of smooth muscle cells (α-SMA) in aortic root plaques, scale bar is 20 μm. Masson staining detection of collagen in aortic root plaques; blue: collagen fibers; scale bar is 100 μm. **(B)** Immunohistochemical detection of smooth muscle cells (α-SMA) in LCCA plaques with positive staining in brown color. Scale bar is 100 μm. Sirius red staining to detect collagen in LCCA plaques, fiber cap (FC) is indicated by arrows, scale bar is 100 μm. **(C–G)** Quantitative staining results (n = 6). ***P < 0.001, compared with CD group; ^#^ *P* < 0.05, ^##^ *P* < 0.01, ^###^ *P* < 0.001, compared with CD + PLCA group; ^&&&^ *P* < 0.001, compared to HFD group, C-D is by one-way ANOVA, E-G is by unpaired *t*-test.

Subsequently, we analyzed smooth muscle cells and collagen content, both of which are determinants of plaque stability (Figure 3). Compared to the CD group, the CD + PLCA group exhibited a significant 23% reduction in smooth muscle cells within the aortic root plaques (*p* < 0.001), while collagen content remained unchanged. In the HFD group, there was a notable 32% reduction in plaque smooth muscle cells (*p* < 0.001), accompanied by a significant 30% reduction in collagen (*p* < 0.01). The HFD + PLCA group demonstrated a substantial decrease in smooth muscle cells by 42% (*p* < 0.001) and a 26% reduction in collagen (*p* < 0.001). When compared to the CD + PLCA group, the HFD group had a 13% decrease in smooth muscle cells (*p* < 0.001) and a 26% reduction in collagen (*p* < 0.01). The HFD + PLCA group exhibited a 26% decrease in smooth muscle cells (*p* < 0.001) and a 22% reduction in collagen (*p* < 0.001). Both the HFD + PLCA group and PLCA group showed a significant 14% reduction in smooth muscle cells (*p* < 0.001), while the collagen did not show significant changes (Figures 3A, C, D).

Through immunohistochemical staining, we detected the expression of α-SMA and collagen (Sirius red stain) in the LCCA plaque. The results revealed a substantial 79% decrease in smooth muscle cell content in LCCA plaques in the HFD + PLCA group compared to the CD + PLCA group (*p* < 0.001), while there was no significant difference in collagen content in the plaques. However, the thickness of the fibrous cap in the HFD + PLCA group was significantly reduced by 30% (60.34 ± 10.33 μm vs. 48.54 ± 2.75 μm, *p* < 0.05) (Figures 3B, E–G).

### 3.3 Plaque vulnerability

**FIGURE 4:**
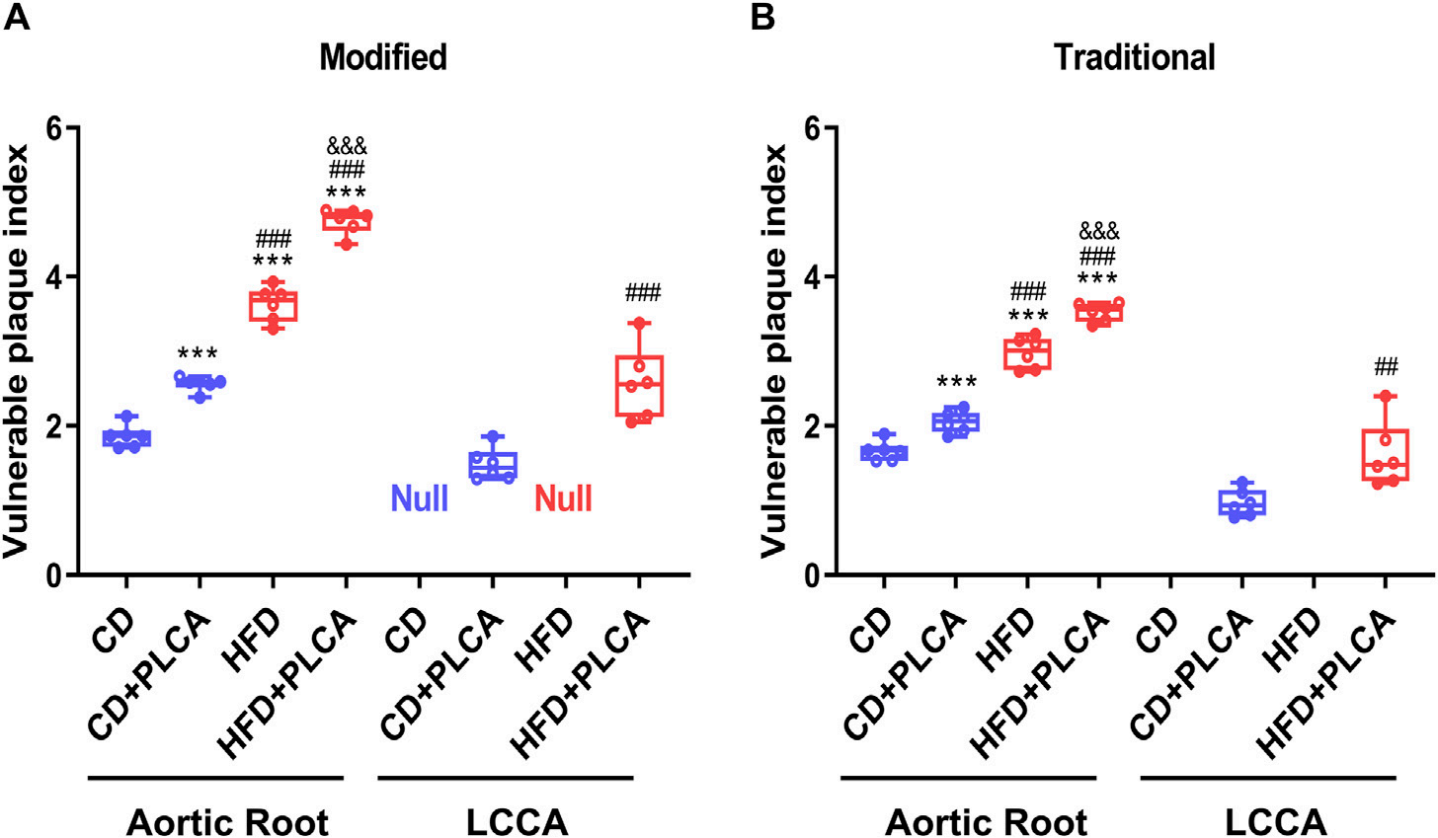
Assessment of Plaque Vulnerability. Box plot showing median and quartiles of vulnerable plaque indexes of aortic root and left common carotid artery (LCCA) plaques in mice evaluated using both the modified **(A)** and traditional **(B)** vulnerability index formulas, n = 6, aortic root is by one-way test, LCCA is by unpaired *t*-test. Notes: VIm, modified vulnerability index; VIt, traditional vulnerability index. ***P < 0.001, compared with CD group; ^##^ *P* < 0.01, ^###^ *P* < 0.001, compared with CD + PLCA group; ^&&&^ *P* < 0.001, compared to HFD group.

The plaque vulnerability index formula initially proposed by Shiomi was refined, taking into account additional variables including VEGF and MMP-9, alongside lipid and macrophage content. This yielded a revised vulnerability index (VI_m_), although Shiomi’s original formula (VI_t_), which only takes into account lipid and macrophage content, was also employed for the assessment of plaque vulnerability in AS mice (Figure 4).

Comparative analysis of results obtained via the revised vulnerability index (VI_m_) revealed a significant increase in the aortic root plaque vulnerability index in the CD + PLCA (2.57 ± 0.10, *p* < 0.001), HFD (3.63 ± 0.23, *p* < 0.001), and HFD + PLCA groups (4.75 ± 0.17, *p* < 0.001), by 38%, 96%, and 1.6-fold respectively, compared to the CD group (1.86 ± 0.15) (Figure 4A). Moreover, the HFD and HFD + PLCA groups had a 42% and 85% higher aortic root vulnerability index than the CD + PLCA group (*p* < 0.001). The HFD + PLCA group also exhibited a 31% increase in the susceptibility index over the HFD group (*p* < 0.001), and a 74% increase in the left carotid plaque susceptibility index (2.58 ± 0.48) over the CD + PLCA group (1.48 ± 0.22) (*p* < 0.001).

These findings underscore the plausibility and effectiveness of the modified formula in augmenting the assessment of plaque vulnerability, as they align with the vulnerability index proposed by Shiomi (Figure 4B). The data suggest that both high-fat diet and PLCA can intensify the vulnerability of AS plaques, with a synergistic effect evident when both factors are present. The vulnerability index ranked as follows: HFD + PLCA group > HFD group > CD + PLCA group > CD group.

These patterns are consistent with the vulnerability index proposed by Shiomi (Figure 4B), thus establishes a basis for further exploration into the utility of refined formulas in vulnerable plaque assessment. The findings indicate a significantly higher degree of plaque vulnerability in the aortic root compared to the LCCA in the same mice. Table 1 summarizes the major measurements across the three additional groups, and Table 2 compares the destabilizing vs. stabilizing components of the plaques.

**TABLE 1:** Summary of lesions in HFD and PLCA apoE^−/−^ mice.

	CD	CD + PLCA	HFD	HFD + PLCA
Vascular diameter	●●●	●●	●●●	●
Velocity	●●●	●	●●	-
Whole aorta Lesion area	●	●●	●	●●●
Aortic root lesion area	●	●●	●●	●●●
Netrocie core	●	●	●●	●●●
LCCA lesion area	-	●●	-	●●●

• low •• medium ••• high, - no visible plaque.

LCCA, left common coronary artery; CD, chow diet; HFD, high-fat diet.

**TABLE 2:** Summary of the plaque components in HFD and PLCA apoE^−/−^ mice.

	CD	CD + PLCA	HFD	HFD + PLCA	Function
SMC	**+++/−**	**++/▲▲▲**	**++**	**+/▲**	stability
Collagen	**+++/−**	**++/▲▲▲**	**+/−**	**+/▲▲▲**	stability
Macrophages	**+/−**	**++/▲**	**++/−**	**+++/▲**	instability
MMP9	**+/−**	**++/▲▲**	**++/−**	**+++/▲▲▲**	instability
VEGF	**+/−**	**++/▲▲**	**++/−**	**+++/▲▲**	uncertain
Lipid	**+/−**	**+/▲▲**	**++/−**	**+++/▲▲▲**	instability

+/**▲** low ++/**▲▲** medium +++/**▲▲▲** high; + aortic root, **▲** LCCA.

- no visible plaque.

LCCA, left common coronary artery; CD, chow diet; HFD, high-fat diet.

### 3.4 Correlation between GCX expression and plaque vulnerability

#### 3.4.1 Positive correlation between SDC1 and plaque vulnerability in AS plaques

**FIGURE 5:**
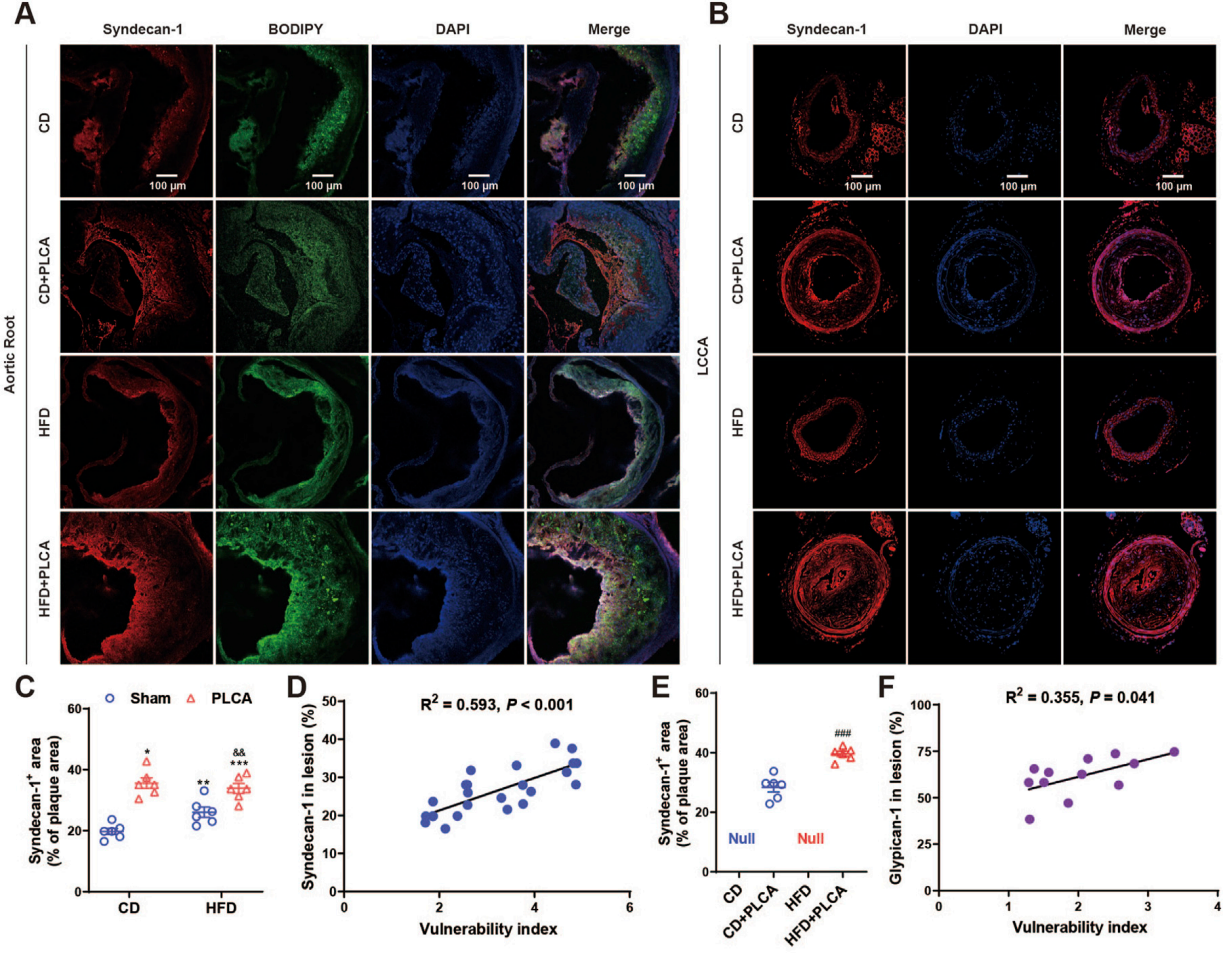
Syndecan-1 staining in AS plaque sections. **(A)** Immunofluorescence detection of syndecan-1 and BODIPY expression in frozen sections of aortic root plaques. Scale bar represents 100 μm. **(B)** Immunofluorescence detection of syndecan-1 expression in LCCA plaques of AS mice. The scale bar represents 100 μm. **(C)** Quantitative analysis of Syndecan-1 staining in aortic root plaques (n = 6, one-way ANOVA). **(D)** Correlation analysis of syndecan-1 expressed in aortic root plaques with plaque vulnerability index, pearson correlation analysis. **(E)** Quantitative analysis of syndecan-1 staining in LCCA plaques (n = 6, unpaired *t*-test). **(F)** Correlation analysis of syndecan-1 expressed in LCCA plaques with plaque vulnerability index, pearson correlation analysis. ^###^ *P* < 0.001, compared with CD + PLCA group.

To investigate the relationship between GCX and plaque vulnerability, we performed immunofluorescence staining of SDC1 on the aortic root and LCCA (Figure 5). The results revealed that SDC1 was predominantly located in the fibrous cap region of the plaques, and its expression was significantly increased by 80% in the CD + PLCA group (*p* < 0.05), 33% in the HFD group (*p* < 0.01), and 73% in the HFD + PLCA group (*p* < 0.001) compared to the CD group (Figure 5A). There was no statistically significant difference in SDC1 expression in the plaques between the HFD and HFD + PLCA groups compared to the CD + PLCA group. However, SDC1 expression was significantly elevated by 30% in the HFD + PLCA group compared to the HFD group (*p* < 0.01) (Figure 5C).

We also performed SDC1 staining on the LCCA plaques using immunofluorescence (Figure 5B). The results showed that SDC1 was mainly localized in the fibrous cap and lipid core regions of the plaques. The expression of SDC1 was significantly increased by 40% in the HFD + PLCA group compared to the CD + PLCA group (*p* < 0.001) (Figure 5E). Moreover, both SDC1 expression in the aortic root plaques (*R* ^2^ = 0.593, *p* < 0.001) and LCCA plaques (*R* ^2^ = 0.523, *p* = 0.008) were positively associated with the vulnerability index (Figures 5D,F).

These findings suggest that an upregulation of SDC1 expression in the plaques is associated with increased plaque vulnerability. Furthermore, the positive correlation between SDC1 expression and the vulnerability index indicates that SDC1 may play a significant role in the development of vulnerable plaques.

#### 3.4.2 Serum SDC1 as an independent predictor of high-risk vulnerable plaque

**FIGURE 6:**
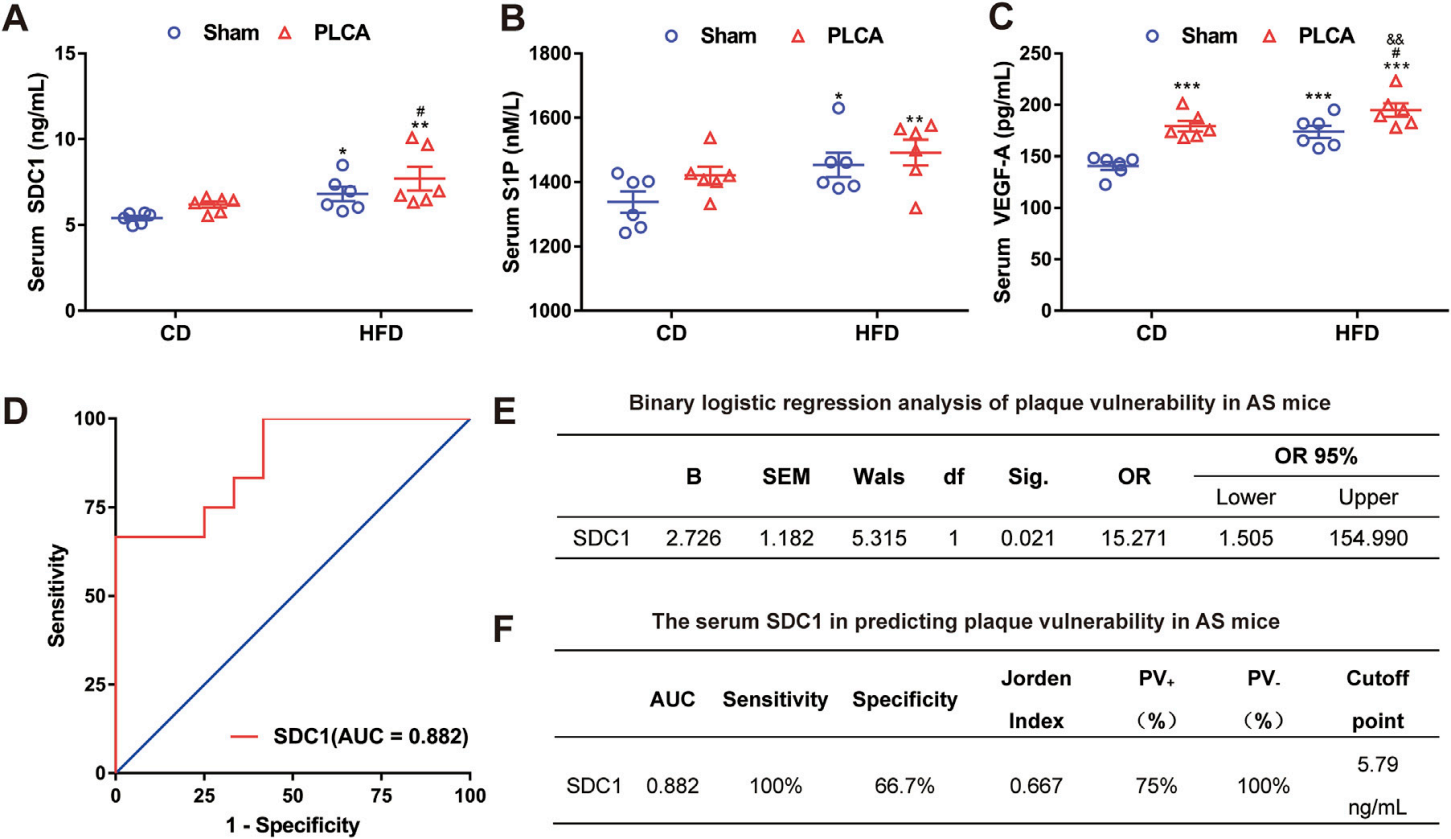
Serum SDC1 is an independent predictor of high-risk vulnerable plaque. **(A)** Serum SDC1; **(B)** S1P; **(C)** VEGF-A was detected using ELISA after 16 weeks of modeling, n = 6, one-way ANOVA. **(D)** ROC curve. **(E)** Multifactor binary logistic regression analysis was performed on serum SDC1, S1P and VEGF-A. **(F)** Receiver operating characteristic (ROC) curve analysis was used to evaluate the diagnostic accuracy of the variables. The Jorden index, calculated as sensitivity minus (1 - specificity), was used to determine the optimal cut-off point on the ROC curve. The area under the ROC curve (AUC) was utilized to assess the clinical prediction value of SDC1. Notes: AUC, area under the curve; PV+, positive predictive value; PV-, negative predictive value. *P < 0.05, *P < 0.01, ***P < 0.001, compared with CD group; ^#^ *P* < 0.05, compared with CD + PLCA group; ^&&^ *P* < 0.01, compared to HFD group.

Levels of SDC1, S1P, and VEGF-A in the serum were measured using ELISA (Figures 6 A–C). SDC1 levels were significantly higher in the HFD group (6.81 ± 0.93 ng/mL, *p* < 0.05), the HFD + PLCA group (7.72 ± 1.56 ng/mL, *p* < 0.01), but were not significantly altered in the CD + PLCA group (6.20 ± 0.40 ng/mL) compared to the CD group (5.41 ± 0.29 ng/mL). SDC1 levels were significantly higher in the HFD + PLCA group compared to the CD + PLCA group (*p* < 0.05), whereas no significant increase was observed in the HFD group. There was no statistically significant difference in SDC1 levels between the HFD + PLCA and the HFD groups.

To delve deeper into potential factors affecting atherosclerotic plaque vulnerability, the vulnerability index (VI) was used to categorize aortic plaque vulnerability into two groups (VI > 2.99 as “1” and VI ≤ 2.99 as “0”) and differences among AS mice were assessed. Binary logistic regression analysis was performed to examine differing correlation indexes among AS mice. The analysis revealed that serum SDC1 levels independently associated with the occurrence of plaque vulnerability in AS mice (*p* < 0.05). The Receiver Operating Characteristic (ROC) curve analysis was performed using the susceptibility index (VI) as the state variable, sensitivity as the *y*-axis and 1-specificity as the *x*-axis (Figure 6D). Multifactor binary logistic regression analysis was performed on serum SDC1, S1P and VEGF-A, results shown that SDC-1 [OR = 15.271(CI = 1.050, 154.990), *p* = 0.021)] acts as an independent risk factor for plaque vulnerability in AS mice (Figure 6E).

The Jorden index was computed and the critical value corresponding to the maximum Jorden index on the ROC curve was identified as the optimal cutoff point (Figure 6F). The area under the curve (AUC) of serum SDC1 was determined to be 0.882, significantly different from the reference line (*p* = 0.001), suggesting moderate accuracy of serum SDC1 in predicting plaque vulnerability in AS mice (0.7 < AUC ≤0.9, Figure 6F). The optimal diagnostic point (cutoff point) for serum SDC1 was 5.79 ng/mL, with a sensitivity of 100% when serum SDC1 > 5.79 ng/mL, and a specificity of 66.7% when serum SDC1 < 5.79 ng/mL. The positive predictive value (PV_+_) was 75% and the negative predictive value (PV_−_) was 100% (Figure 6F), indicating the predictive value of serum SDC1 for AS plaque vulnerability.

## 4 Discussion

We presently evaluated the vulnerability of atherosclerotic plaques and investigated the correlation between GCX and vulnerability in atherosclerotic mice. Our findings underscore the importance of serum SDC1 in plaque vulnerability, and the modified vulnerability index presents a viable tool for assessing high-risk plaques in atherosclerosis. Furthermore, our results highlight the link between GCX injury and atherosclerotic disease, suggesting the potential utility of circulating SDC1 levels as a prognostic marker for coronary artery disease.

Studies have shown that estrogen can reduce the burden of atherosclerotic plaques (Cignarella et al., 2024). Apolipoprotein E-deficient (ApoE^−/−^) male mice are predisposed to atherosclerosis (Zhao et al., 2020). Thus, male C57BL/6-ApoE^−/−^ mice were selected to eliminate the confounding effects of estrogen. In addition, our study employed a dual approach: a high-fat diet alongside partial ligation of the left carotid artery (PLCA), to precipitate carotid atherosclerosis in ApoE^−/−^ mice. Partial ligation was executed near the bifurcation of the common carotid artery—a strategic choice informed by the site’s unique anatomical and hemodynamic characteristics, which predispose it to atherosclerosis (Chen et al., 2023). Partial ligation ostensibly disrupts blood flow, thereby fostering atherosclerosis. Besides, compared to C57BL/6 mice, ApoE^−/−^ mice on a standard diet naturally develop hypercholesterolemia and may, between 11–21 weeks, show signs of smaller, absent foam cells or calcified deposits in the carotid arteries or aorta. Carotid intima-media thickness (IMT) measurements, detected via Doppler ultrasound, along with the presence of atheromatous plaque, correlate with cardiovascular risk factors, cardiovascular event rates, and mortality (Willeit et al., 2020). To economize on animal usage and mitigate unpredictable variables, we utilized small animal ultrasound to monitor changes in carotid IMT in ApoE^−/−^ mice. In our study, we used well-examined ApoE^−/−^ mice to establish an atherosclerosis model by ligating the carotid artery, inducing low flow velocity and shear stress. ApoE^−/−^ mice fed a high-fat diet for 16 weeks exhibited significant stenosis and reduced flow velocity in their left common carotid artery. Concurrently, there were notable increases in total cholesterol and LDL serum levels, and our data identified considerable plaque formation and lipid deposition in the aortic root and carotid intima, as evidenced by oil red O and HE staining. These plaques appeared to represent mid to later-stage disease progression (Figure 1).

The aim of this part of the study was to evaluate the vulnerability of atherosclerotic plaques in order to facilitate subsequent investigations into the correlation between GCX and vulnerability. ApoE^−/−^ mice fed a regular chow diet did not exhibit necrotic core formation, had thicker fibrous caps, and less macrophage infiltration, indicating that aortic root plaques were not susceptible to rupture. Previous studies have shown that ApoE^−/−^ mice fed a regular chow diet require more than 20 weeks for features like fibrous cap to manifest within the plaque (Nakashima et al., 1994). Therefore, in our study, we created a vulnerable atherosclerosis model using a high-fat diet and/or partial ligation of the left common carotid artery (PLCA).

Historically, attempts to predict cardiovascular events have primarily concentrated on either stable or unstable plaque components. Given the limitations of individual histological features in identifying plaques susceptible to rupture, a composite of several features is often required. Consequently, we calculated a vulnerability index (VI) that incorporates histological features to capture the predominant markers for stable plaque elements (macrophages, hemorrhage, and lipid content) and unstable components (smooth muscle cells and collagen content). The balance between these elements determines potential plaque rupture. The VI methodology has been replicated with slight modifications in animal studies and in research examining human plaque composition using ultrasound, magnetic resonance imaging, and studies of human plaque cell metabolism (Shiomi et al., 2001; Hartwig et al., 2015; Erlöv et al., 2016; Tomas et al., 2018; Edsfeldt et al., 2022).

Macrophages, with their two primary phenotypes M1 and M2, play pivotal roles in the pathogenesis of atherosclerosis (Barrett, 2020). The M1 phenotype is characterized by pro-inflammatory properties and its ability to form foam cells, a hallmark of early atherosclerotic lesions. Conversely, the M2 phenotype, while also generating inflammatory mediators, is associated with resistance to plaque progression. Gene and protein expression analysis supported the presence of M1 and M2 markers at higher concentrations in macrophages from symptomatic and asymptomatic individuals, respectively, in a large-scale study (de Gaetano et al., 2016). In this study, we used CD68 as a marker for macrophages, and the utility of employing the M1/M2 ratio as a means to calculate plaque vulnerability is a proposition that warrants further exploration.

In recent, it was demonstrated that immature neovascularization, characterized by a lack of support, high brittleness, and permeability, can trigger intraplaque hemorrhage upon rupture (Parma et al., 2017). This leads to leukocyte and platelet infiltration into the plaque and the release of free cholesterol from apoptotic erythrocytes, which transform into foam cells after macrophage phagocytosis (Wang et al., 2018). VEGF is vital in increasing vascular permeability and inducing neovascularization (Melincovici et al., 2018). Intraplaque hemorrhage can also stimulate MMPs secretion (Suzuki et al., 2018). Increased MMPs activity weakens the tensile strength of the plaque fibrous cap, allowing the lipid core within the enlarged plaque to destabilize the vessel and ultimately cause plaque rupture (Liu et al., 2014). Therefore, VEGF and MMP-9 were incorporated into the modified vulnerability index calculation formula. The feasibility of the vulnerability index calculation was confirmed by comparing two different vulnerability index formulas to evaluate high-risk plaques in patients who died of coronary heart disease (Supplementary Figure S1). Prominent plaques were observed in the human aorta, and lipid staining revealed a significant accumulation of lipids and macrophages in these plaques. As outlined in the current medical literature carried out by the traditional methods, the vulnerability index values for high-risk human atherosclerotic plaques ranging from 3.88 (1.72–8.77), which are associated with the risk of cardiovascular events (Goncalves et al., 2021). In the limited lesion we evaluated, the differences in vulnerability index among the groups obtained by both formulas were consistent. The modified methods show a distinct separation among the groups, as depicted in the box plot (Figure 4), which is beneficial for identifying and analyzing the differences between the groups. Conversely, the traditional method exhibits an obvious overlap in its extreme values, which impedes the accurate identification and interpretation of the variations among the different groups. Specifically, the maximum values of the CD and HFD groups, as determined by the traditional method, exceeded the minimum values of the CD + PLCA and HFD + PLCA groups, respectively, suggesting that the traditional method may not be as effective as the modified method in distinguishing plaques induced by different conditions and etiologies (e.g., diet factors vs. mechanical injury). In fact, there are various risk factors for plaque formation in human *in vivo*, and the modified method might be more suitable for identifying and classifying vulnerable plaques influenced by a variety of distinct risk factors. Nevertheless, further research is essential to determine the applicability of this assessment in more complex plaques scenarios.

In the aortic lesion and the left common carotid plaque region, our results revealed that, the vulnerability index ratio (macrophages + lipid deposits + VEGF + MMP-9)/(smooth muscle cells + collagen fibers) was elevated. Following a high-fat diet for 16 weeks, ApoE^−/−^ mice in the HFD and HFD + PLCA groups exhibited extensive lipid and macrophage accumulation in the aortic root plaques, along with a large lipid necrotic core and cholesterol crystals. These plaques also showed diminished collagen and smooth muscle cell expression, thinning of the fibrous cap, thus presenting a markedly vulnerable plaque phenotype. The HFD + PLCA group also exhibited increased lipid composition (macrophages and/or extracellular lipid deposits) throughout the aortic plaque and fibrous cap region of the left common carotid plaque. The incidence of vulnerable plaques in the aortic root lesions was significantly higher in the HFD and HFD + PLCA groups compared to the CD group. These findings underscore that importance of reducing the lipid component in the fibrous cap for plaque stabilization. Conversely, in the CD + PLCA group, the percentage area of smooth muscle cells or fibromuscular component was increased, and the predisposition to atherosclerotic plaque formation was reduced compared to the CD group. This is consistent with the understanding that a reduction in the muscle fiber component of the fibrous cap is correlated with plaque instability (Wang et al., 2015).

The injury and shedding of GCX have been reported in various disease states, including sepsis, ischemia-reperfusion injury, acute respiratory distress syndrome, and cardiogenic shock (Frydland et al., 2018). The GCX injury is believed to result from various vascular stresses, such as oxidized low-density lipoprotein (ox-LDL), shear instability (Haroon et al., 2022), and hypertonia, suggesting a close link between GCX deterioration, the development of atherosclerosis, and subsequent cardiovascular disease. Numerous clinical studies have explored the association between GCX injury and atherosclerotic disease. For instance, Miranda et al., 2016 demonstrated that SDC1 levels were elevated and persisted for 12 h following the onset of acute coronary syndrome, indicating the involvement of acute GCX injury in this syndrome development. Elevated serum SDC1 levels were also noted in patients with stress cardiomyopathy (Nguyen et al., 2017), suggesting acute GCX injury. Serum SDC1 were elevated with the severity of vulnerability, a trend not observed with glypican-1 (Supplementary Figure S2), suggesting the shedding of SDC1 in progression of atherosclerotic plaque. We previously demonstrated that MMPs play critical role in the degradation of SDC1 under serum-free conditions (Zeng et al., 2014). Additionally, we observed that the endothelial GCX does not collapse after heparan sulfate degradation by heparanase III treatment (Zeng et al., 2012). These findings set the stage for a deeper exploration into the dynamics of GCX components following SDC1 degradation and the complex mechanisms that regulate the degradation and deposition of SDC1 within atherosclerotic plaques.

In recent decades, several studies have measured circulating S1P levels to determine its role as a prognostic marker for the development of CAD (Sattler et al., 2010; Schielke et al., 2023). Patients with CAD have been found to have reduced S1P levels in HDL compared to healthy individuals (Sattler et al., 2010). Lower HDL-bound plasma S1P levels have also been observed in patients with multivessel disease compared to those with single-vessel disease, indicating a negative correlation between CAD severity and plasma-bound S1P levels (Sattler et al., 2014). Our ELISA results showed high S1P expression in the serum of AS model mice, with the highest levels observed in the HFD + PLCA group. In agreement with our findings, Keul et al. investigated the effect of persistently high S1P levels on atherosclerosis in cholesterol-fed ApoE^−/−^ mice over 12 weeks and found that high endogenous S1P levels promote atherosclerosis, impair cholesterol efflux, and lead to true plaque rupture (Keul et al., 2022). We previously demonstrated that S1P plays a protective role by suppressing the shedding of sydnecan-1 and preserving the integrity of the endothelial GCX (Zeng et al., 2014). However, the simultaneous elevation of both serum S1P and SDC1 challenges the role of S1P in atherosclerosis.

The association between vascular neovascularization and the growth and stabilization of atherosclerotic plaques has raised questions about the therapeutic use of VEGF for vascularization (Moreno et al., 2006). Although clinical trials involving hundreds of patients have not shown any indication that VEGF promotes or accelerates atherosclerosis and its clinical outcomes, the role of VEGF in atherogenesis remains uncertain due to inconsistent results from animal studies. Schüler et al. (2018) found that high-fat feeding in ApoE^−/−^ mice resulted in elevated plasma VEGF expression, which is in line with our results showing significantly elevated serum VEGF levels in AS model mice. Another study comparing serum levels of GCX in patients with type 1 diabetes mellitus (T1DM) with and without renal failure found significantly elevated levels of SDC1 and VEGF in the plasma circulation of patients with nephropathy (Kolseth et al., 2017). Plasma SDC1 was also found to correlate well and independently with renal (plasma creatinine and plasma urea) and endothelial function parameters (plasma VEGF) in a rat kidney transplantation model (Adepu et al., 2015).

This research has extended our understanding of atherosclerotic plaque vulnerability by employing the vulnerability index (VI) to categorize aortic plaque vulnerability into two distinct classes in AS mice. Notably, it was discovered that serum SDC1 levels rather than S1P and VEGF are independently linked to the incidence of plaque vulnerability. Through the implementation of a ROC curve analysis and the calculation of the Jorden index, the study has also indicated the significant role of serum SDC1 as a predictive biomarker for plaque vulnerability, exhibiting moderate precision. The optimal diagnostic point was determined to be 5.79 ng/mL for serum SDC1, offering both strong sensitivity and specificity, thereby underlining the predictive capability of serum SDC1 for AS plaque vulnerability. This study contributes significantly to the field of AS by providing a novel biomarker, serum SDC1, that shows potential in predicting and assessing plaque vulnerability in AS mice. However, further studies are required to validate these findings and to explore the therapeutic potential of these factors in the prevention and treatment of atherosclerosis and associated cardiovascular diseases.

## 5 Significance

This study highlights the significance of syndecan-1 in plaque vulnerability and provides valuable insights into the formation of high-risk vulnerable plaques in atherosclerosis. These findings have substantial implications for cardiovascular disease management, as early identification of vulnerable plaques can lead to timely interventions and prevent life-threatening events. Moreover, the exploration of syndecan-1 as a biomarker offers a promising tool for risk stratification and personalized treatment approaches.

## Data Availability

The original contributions presented in the study are included in the article/Supplementary Material, further inquiries can be directed to the corresponding author.

## Supplementary material

SUPPLEMENTAL FIGURE S1 Evaluation of vulnerability to human aortic plaque. Left: Representative images of aortic staining for VEGF, CD68, MMP-9, α-SMA immunofluorescence and Masson trichrome staining. Scale bar = 100 μm; Right: Calculation results of the vulnerability index. ***P* < 0.001 by unpaired t-test.

SUPPLEMENTAL FIGURE S2 Serum glypican-1 in AS mice. **P* < 0.01, ****P* < 0.001, compared with CD group (n = 6, one-way ANOVA); ###*P* < 0.0001, compared with CD+PLCA group; ^&&&^ *P* < 0.001, compared to HFD group.
