# Supplementary figures and images for: Syndecan-1 as a predictor of vulnerable atherosclerotic plaques
Source: Front Cell Dev Biol. 2024 Aug 8;12:1415788. doi: 10.3389/fcell.2024.1415788 (PMC11338802; doi:10.3389/fcell.2024.1415788)

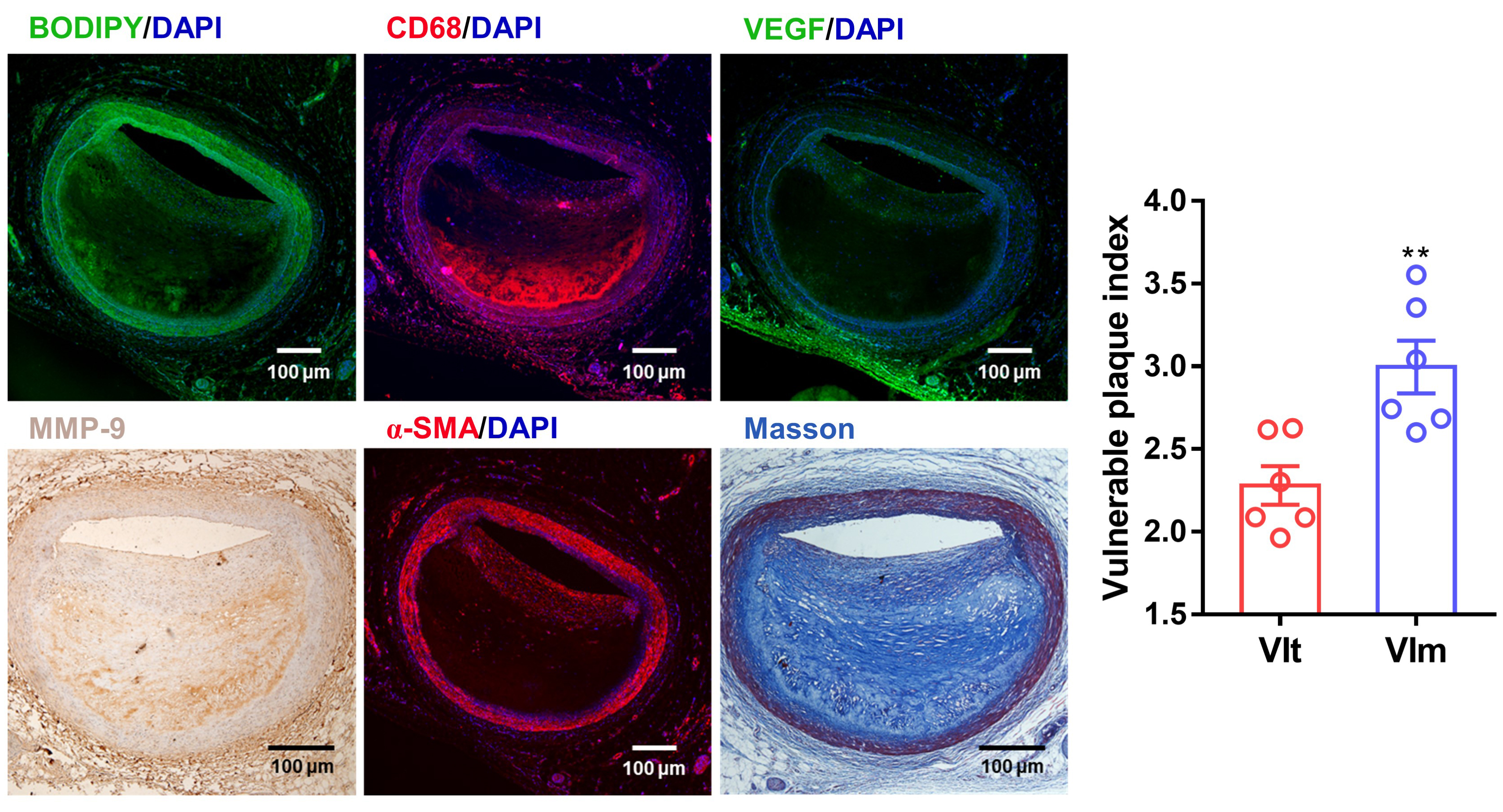

Supplement: Supplementary file 1 [file Image1.JPEG]

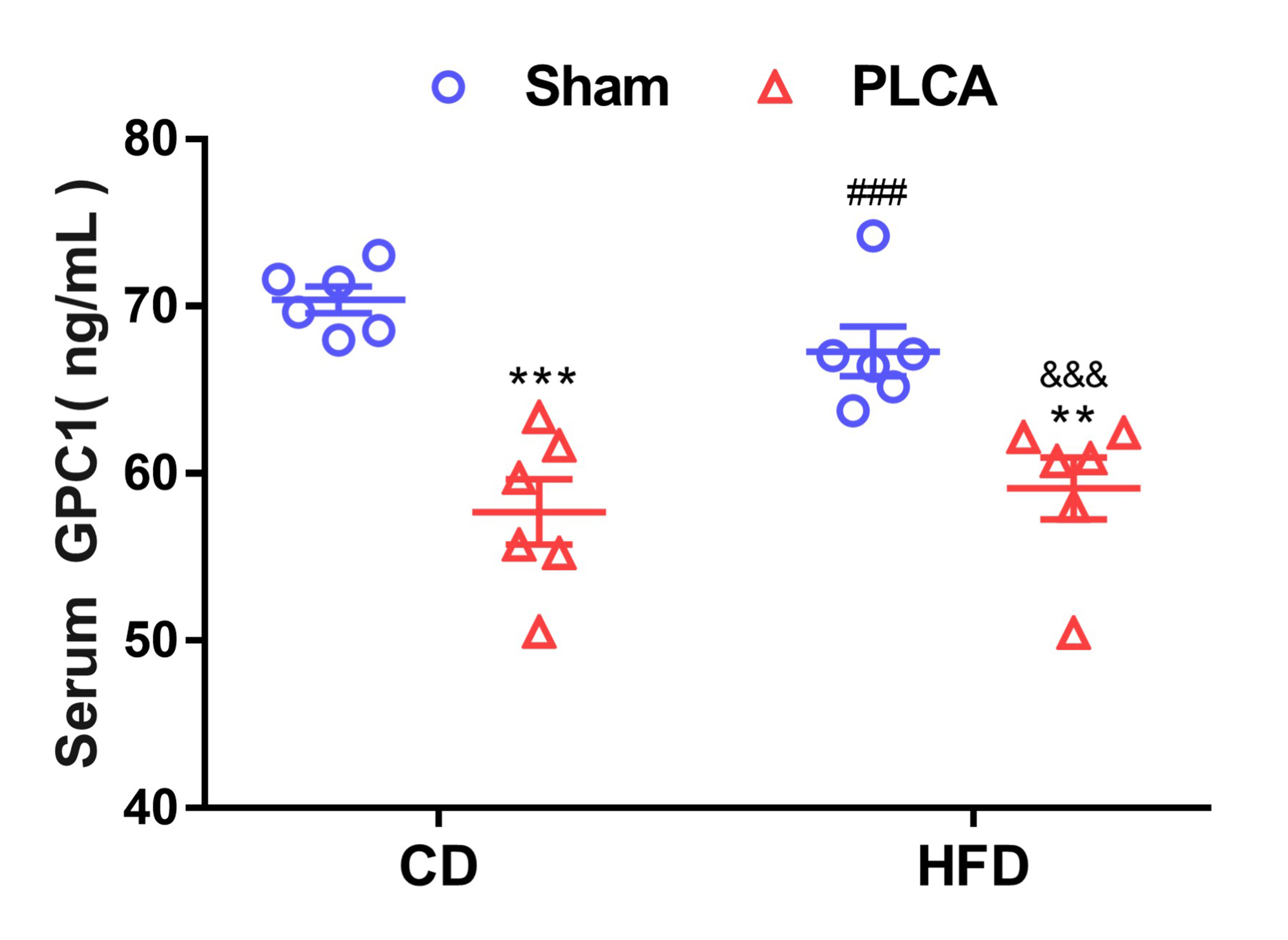

Supplement: Supplementary file 2 [file Image2.JPEG]
